# A Transformer-Based Ensemble Framework for the Prediction of Protein–Protein Interaction Sites

**DOI:** 10.34133/research.0240

**Published:** 2023-09-27

**Authors:** Minjie Mou, Ziqi Pan, Zhimeng Zhou, Lingyan Zheng, Hanyu Zhang, Shuiyang Shi, Fengcheng Li, Xiuna Sun, Feng Zhu

**Affiliations:** ^1^ College of Pharmaceutical Sciences, The Second Affiliated Hospital, Zhejiang UniversitySchool of Medicine, National Key Laboratory of Advanced Drug Delivery and Release Systems, Zhejiang University, Hangzhou 310058, China.; ^2^ Innovation Institute for Artificial Intelligence in Medicine of Zhejiang University, Alibaba-Zhejiang University Joint Research Center of Future Digital Healthcare, Hangzhou 330110, China.

## Abstract

The identification of protein–protein interaction (PPI) sites is essential in the research of protein function and the discovery of new drugs. So far, a variety of computational tools based on machine learning have been developed to accelerate the identification of PPI sites. However, existing methods suffer from the low predictive accuracy or the limited scope of application. Specifically, some methods learned only global or local sequential features, leading to low predictive accuracy, while others achieved improved performance by extracting residue interactions from structures but were limited in their application scope for the serious dependence on precise structure information. There is an urgent need to develop a method that integrates comprehensive information to realize proteome-wide accurate profiling of PPI sites. Herein, a novel ensemble framework for PPI sites prediction, EnsemPPIS, was therefore proposed based on transformer and gated convolutional networks. EnsemPPIS can effectively capture not only global and local patterns but also residue interactions. Specifically, EnsemPPIS was unique in (a) extracting residue interactions from protein sequences with transformer and (b) further integrating global and local sequential features with the ensemble learning strategy. Compared with various existing methods, EnsemPPIS exhibited either superior performance or broader applicability on multiple PPI sites prediction tasks. Moreover, pattern analysis based on the interpretability of EnsemPPIS demonstrated that EnsemPPIS was fully capable of learning residue interactions within the local structure of PPI sites using only sequence information. The web server of EnsemPPIS is freely available at http://idrblab.org/ensemppis.

## Introduction

Protein–protein interaction (PPI) plays a fundamental role in numerous cellular functional progresses [1–5]. PPI sites refer to the interfacial residues of proteins that are involved in these interactions, and the identification of PPI sites is of utmost importance for unraveling the mysteries of cell processes and promoting the development of new drugs [6–8]. Experimental approaches for identifying PPI sites, including affinity purification coupled to mass spectrometry [9,10], coimmunoprecipitation [11,12] and 2-hybrid screening [13,14], face challenges due to their intricate and time-consuming procedures [15–18]. Therefore, the development of efficient computational methods to accelerate the identification of PPI sites is of vital importance [19–22].

So far, various computational methods have been developed for predicting PPI sites, which can be categorized into 2 mainstream strategies [23]. The first strategy involves docking methods that predict pairwise interaction sites and rely on the structural information of both interacting proteins [24,25]. In contrast, the second strategy focuses on predicting putative interaction sites within individual isolated proteins, without requiring any knowledge of the partner proteins [26]. The latter strategy holds great research importance since the structure of protein complex or the information on partner protein may not be available, and therefore has spawned a series of machine learning-based tools to perform partner-independent prediction of PPI sites in a more general paradigm [17]. These tools were built to learn and extract information that determines PPI, and were broadly categorized into sequence-based and structure-based according to the type of input data [27–29]. Some tools encode residues from the primary sequence and output the probability of being PPI sites [30], such as SPRINGS [31], SCRIBER [32], ProNA2020 [33], and DELPHI [34]. Others leverage structural information to identify PPI sites, such as secondary structure and residue contact map. Prominent examples in this category include SPPIDER [28], DeepPPISP [22], EGRET [23], and GraphPPIS [35]. Recently, several methods utilized geometric deep learning to capture structural surface features for PPI sites prediction, including PInet [36], MaSIF-site [37], ScanNet [38], and PeSTo [39].

However, these methods suffer from the low predictive accuracy or the limited scope of application. Specifically, some methods had a main disadvantage of relatively low prediction accuracy because they only excelled at learning global or local contextual features from primary sequences [22,23,40,41], but failed to leverage local structural features whose information proves to be inextricably linked to PPI sites [23,35,42]. Others achieved improved performance by extracting residue interactions from protein structures, particularly the long-range interactions within local structures, but their application scope and generalization ability were extremely limited for their acute dependence on precise structure information, severe sensitivity to structural errors, and inappropriate use of protein conformation for model training [35,43,44]. Therefore, there is an urgent need to develop a method that integrates comprehensive information to enable accurate identification of PPI sites in the largest scope of whole proteome [45–47].

Herein, a novel transformer-based ensemble method for PPI sites prediction, EnsemPPIS, was therefore proposed, which can capture not only global and local patterns but also residue interactions. EnsemPPIS consists of 2 base models, namely, TransformerPPIS and GatCNNPPIS. The transformer framework in TransformerPPIS is equipped with the ability to learn global features and calculate attention weights between residues, making it possible to capture residue dependencies within local structures, while GatCNNPPIS is capable of learning local contextual features using the gated convolutional networks. EnsemPPIS was thoroughly evaluated on multiple PPI sites prediction tasks and exhibited either superior performance or broader applicability compared with various existing methods. Moreover, pattern analysis based on the interpretability of EnsemPPIS demonstrated that EnsemPPIS was fully capable of learning residue interactions using only primary sequences, thereby improving the performance of PPI sites prediction. A web server of EnsemPPIS was further established, which is freely available at http://idrblab.org/ensemppis. EnsemPPIS is applicable for proteome-wide profiling of PPI sites and expected to provide more insights into protein function research and drug discovery.

## Results and Discussion

### The ensemble framework of EnsemPPIS for predicting PPI sites

**Fig. 1.**
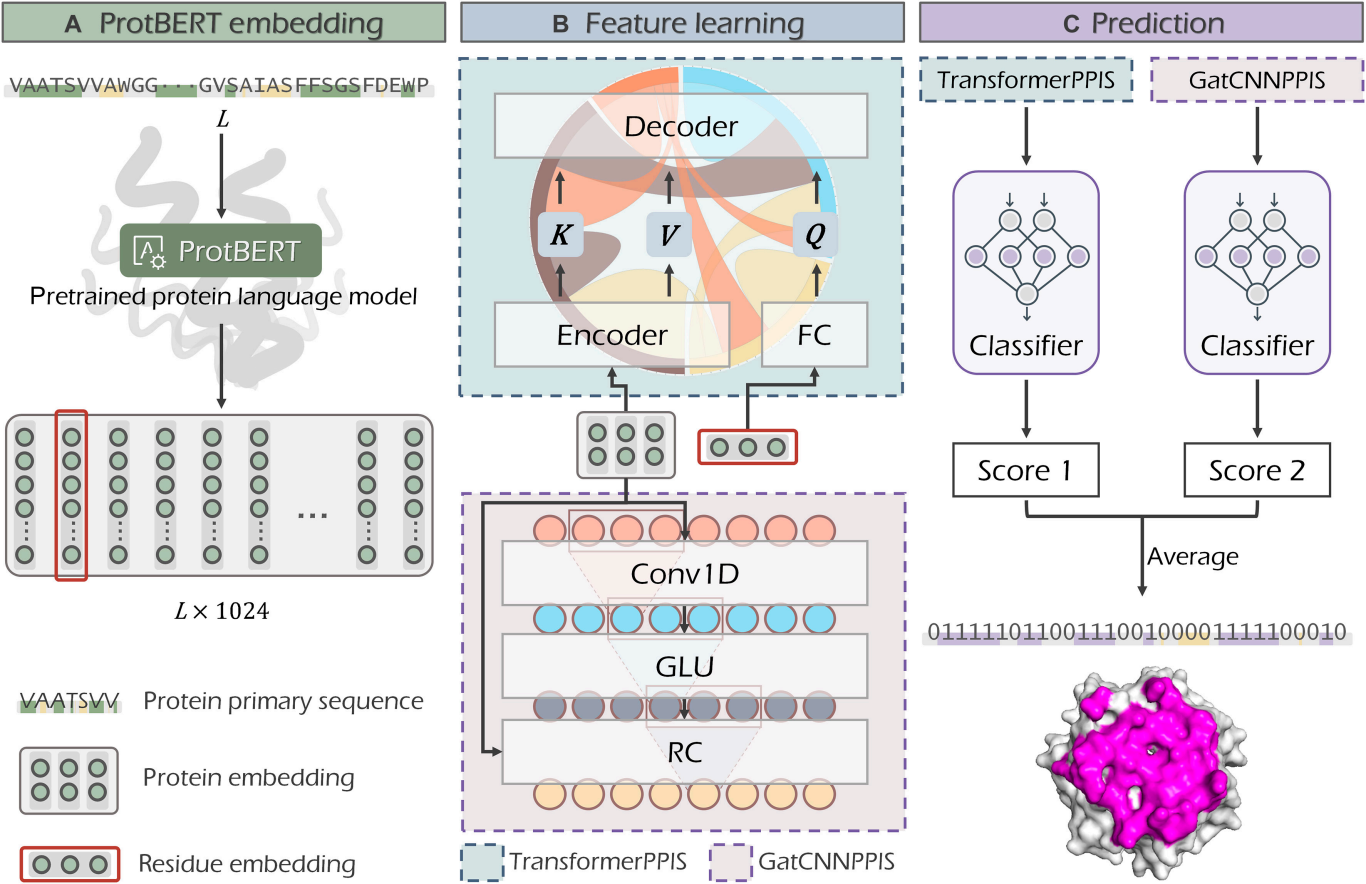
The ensemble learning framework of EnsemPPIS for predicting PPI sites. EnsemPPIS consists of 2 base models (TransformerPPIS and GatCNNPPIS) and functions through 3 steps, including ProtBERT embedding, feature learning and prediction. The average of probability scores output by the 2 base models is considered as the final probability of each residue as a potential PPI site. GLU, gated linear unit; RC, residual connection; FC, fully connected layer.

**Fig. 2.**
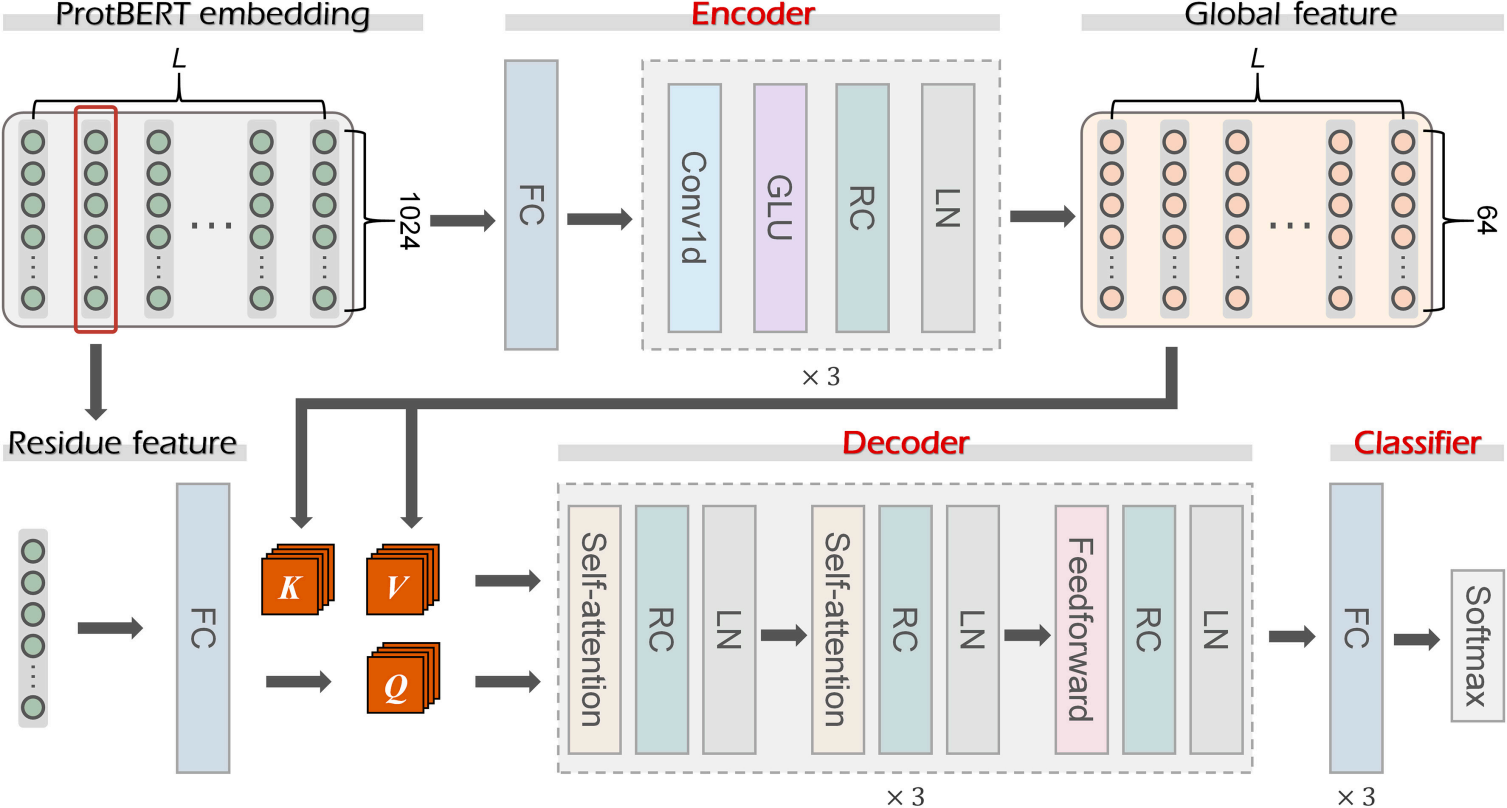
The deep learning architecture of the base model TransformerPPIS in EnsemPPIS. TransformerPPIS is mainly composed of 3 modules: the encoder, the decoder, and the classifier module. The sequence embedding obtained by ProtBERT is first input into the encoder module to extract global feature. Then, the global feature of the protein and the original embedding feature of a specific residue are both input into decoder module. The output of decoder is further passed into the classifier module to generate the probability score of a residue being a potential PPI site. GLU, gated linear unit; RC, residual connection; LN, layer normalization; FC, fully connected layer.

EnsemPPIS functions through 3 steps, including ProtBERT embedding, feature learning, and prediction, as illustrated in Fig. 1. Specifically, proteins are input into ProtBERT, a pretrained protein language model, to obtain the embeddings for residues [48]. Following the embedding, an ensemble learning framework is employed to effectively learn the underlying features, which consists of 2 deep learning base models, namely, TransformerPPIS and GatCNNPPIS. These models leverage the embeddings obtained from ProtBERT for further analysis and prediction of PPI sites. TransformerPPIS can extract residue interaction information and global features of proteins. To extract global features, the protein embeddings are fed into the encoder module. Simultaneously, each residue embedding undergoes a fully connected layer (FC) before being input into the decoder module alongside the global features. Within the decoder, the pairwise residue interactions are extracted using the self-attention mechanism of the transformer algorithm. The concrete architecture of TransformerPPIS is illustrated in Fig. 2, with a more detailed description presented in Materials and Methods. GatCNNPPIS can extract local features from protein embeddings. Specifically, GatCNNPPIS employs gated convolutional networks with residual connections to capture sequential motifs. In this approach, each residue is represented by its local contextual environment, which encompasses a total of 7 residues. Finally, the latent representations generated by TransformerPPIS and GatCNNPPIS are separately fed into the classifier, which consists of several FCs. The classifier utilizes these representations to output the probability score. The average probability score serves as the final probability of each residue being a potential PPI site. In summary, the major characteristic of EnsemPPIS is its ability to extract local and global features, as well as residue interaction information from ProtBERT-embedded proteins based on the ensemble learning framework.

### Leading performance of EnsemPPIS in residue-level prediction

Previous studies have generated multiple datasets preserving experimentally validated PPI sites data, which have been widely utilized in developing computational tools, as displayed in Table S1. We took advantage of these valuable benchmark datasets to train and evaluate EnsemPPIS and made comprehensive comparisons with various existing methods. As a result, EnsemPPIS achieved leading performance in residue-level prediction on *DeepPPISP task* and *DELPHI task*.

(a) *Performance evaluation on DeepPPISP task*

EnsemPPIS, along with 12 other competing methods, was first evaluated and compared on the *DeepPPISP task*, as shown in Table 1. Some of the results were obtained by reproducing the provided source code or utilizing the web server. Meanwhile, for certain methods that employed the same training and test data as the previous work DeepPPISP [22], the results were directly collected from that study to ensure consistency and comparability.

**Table 1.** Comparison of the predictive performance of our proposed methods and other state-of-the-art methods on *DeepPPISP task*. DeepPPISP, EGRET, IntPred, and SPPIDER use protein structural information. DELPHI, DLPred, ISIS, ProNA2020, PSIVER, RF_PPI, SCRIBER, and SPRINGS use protein sequences. TransformerPPIS, GatCNNPPIS, and EnsemPPIS are proposed in this study. All comparison methods are sorted alphabetically. The best results are shown in bold.

Method	ACC	PRE	REC	F1	AUROC	AUPRC	MCC
DeepPPISP ^a^	0.655	0.303	0.577	0.397	0.671	0.320	0.206
DELPHI ^b^	0.667	0.319	0.604	0.418	0.690	0.360	0.236
DLPred ^c^	0.680	0.325	0.577	0.416	0.697	0.380	0.235
EGRET ^b^	0.715	0.358	0.561	0.438	0.719	0.405	0.270
IntPred ^a^	0.672	0.247	0.508	0.332	-	-	0.165
ISIS ^a^	0.622	0.211	0.362	0.267	-	0.240	0.097
ProNA2020 ^c^	**0.741**	0.297	0.229	0.258	-	-	0.106
PSIVER ^a^	0.653	0.253	0.468	0.328	-	0.250	0.138
RF_PPI ^a^	0.598	0.173	0.512	0.258	-	0.210	0.118
SCRIBER ^c^	0.616	0.274	0.569	0.370	0.635	0.307	0.159
SPPIDER ^c^	0.667	0.240	0.315	0.273	0.518	0.235	0.063
SPRINGS ^a^	0.631	0.248	0.598	0.35	-	0.280	0.181
TransformerPPIS	0.681	0.332	0.604	0.429	0.711	0.389	0.253
GatCNNPPIS	0.633	0.306	**0.698**	0.421	0.698	0.369	0.239
EnsemPPIS	0.732	**0.375**	0.532	**0.440**	**0.719**	**0.405**	**0.277**

^a^ Results reported by DeepPPISP. ^b^ Results obtained by reproducing the source code. ProNA2020 only makes binary predictions, and its AUROC and AUPRC are not calculated. ^c^ Results obtained by utilizing the web server.

As a result, EnsemPPIS achieved the highest performance among all evaluated methods, achieving the MCC value of 0.277, AUPRC of 0.405, and F1 of 0.405. These 3 evaluation metrics are the most important ones in the imbalanced task of PPI sites prediction [22]. Specifically, when compared to the state-of-the-art (SOTA) sequence-based method DELPHI, EnsemPPIS achieved a 5.3% improvement in F1, a 12.5% improvement in AUPRC, and a remarkable 17.4% improvement in MCC. Moreover, EnsemPPIS, using only sequence information, exhibited competitive performance even when compared to structure-based methods. In fact, EnsemPPIS slightly outperformed the most recent method, EGRET, in terms of F1 and MCC. The performance of the 2 base models, TransformerPPIS and GatCNNPPIS, was also evaluated. TransformerPPIS exhibited superior performance compared to most of the existing methods, showcasing its effectiveness in leveraging global features and residue interactions from the protein embeddings. On the other hand, GatCNNPPIS achieved strong performance, highlighting its ability to capture local contextual information. Both models demonstrated their efficacy and contributed to the overall success of the EnsemPPIS framework. In general, EnsemPPIS achieved the highest performance, indicating the effectiveness of ensemble learning. Importantly, the PRE value of EnsemPPIS demonstrated an increase compared to that of the base models. This indicated that ensemble learning effectively contributed to controlling the false-positive rate to a certain extent.

**Fig. 3.**
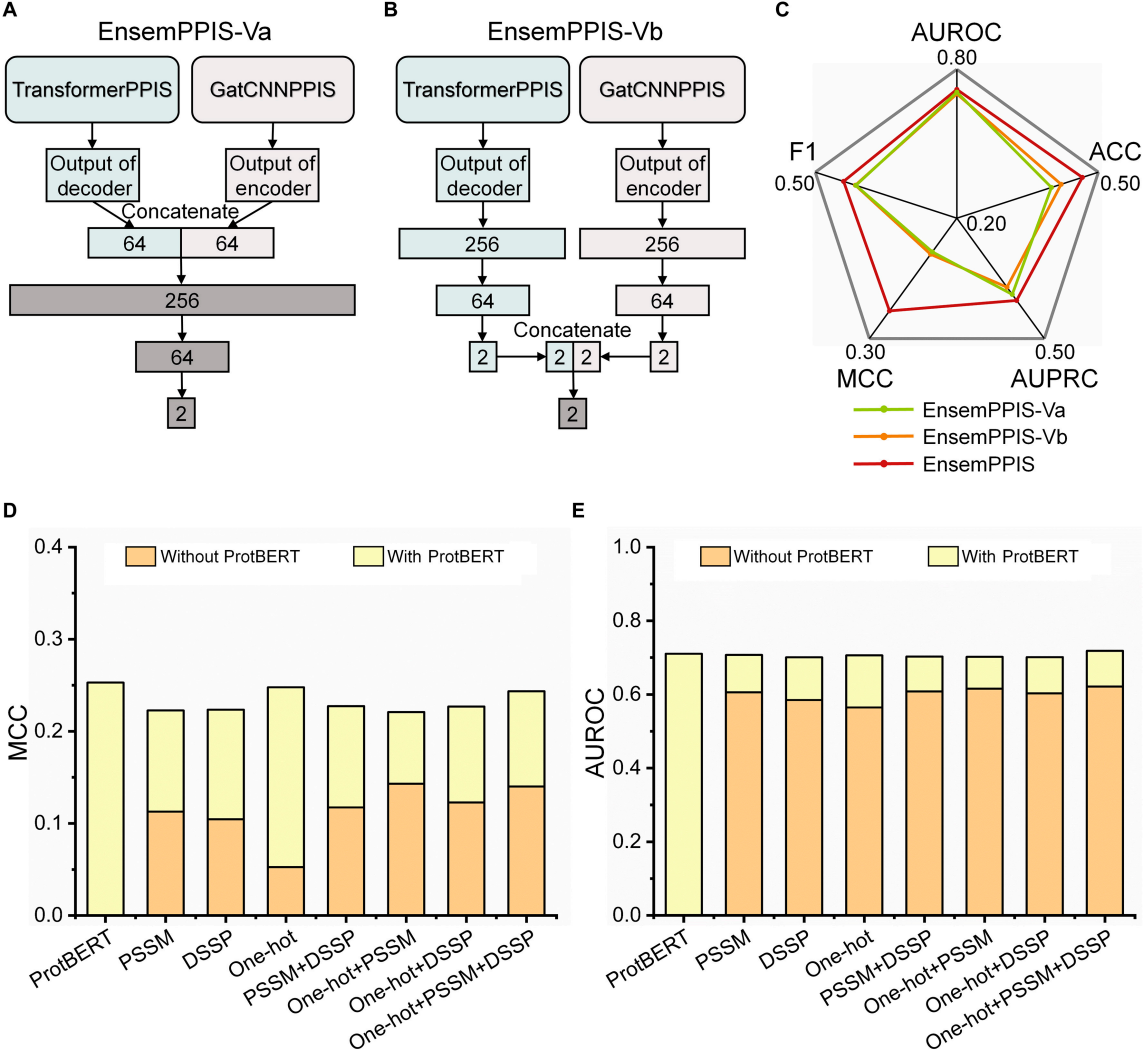
Performance evaluation of EnsemPPIS, its variants, and the base model on the *DeepPPISP task*. (A) Architecture of variant EnsemPPIS-Va. The output of TransformerPPIS’s decoder and the output of GatCNNPPIS’s encoder were concatenated. The concatenated vector was then fed into multiple fully connected layers (FCs). (B) Architecture of variant EnsemPPIS-Vb. The output of TransformerPPIS’s decoder and the output of GatCNNPPIS’s encoder were separately passed through FCs. The resulting 2-dimensional vectors were concatenated and further processed through an FC. (C) Performance comparison of EnsemPPIS, EnsemPPIS-Va, and EnsemPPIS-Vb on various metrics. (D) Matthews correlation coefficient (MCC) of TransformerPPIS using different types of feature. (E) Area under the receiver operator characteristic curve (AUROC) of TransformerPPIS using different types of feature. The orange bars represent the performance without ProtBERT feature, and the yellow bars represent the performance with inclusion of ProtBERT feature.

EnsemPPIS achieves accurate prediction of PPI sites by integrating 2 separately trained base models. To demonstrate the effectiveness of ensemble learning, 2 variants of EnsemPPIS were constructed by combining the 2 base models into a single model for concurrent training, namely, EnsemPPIS-Va and EnsemPPIS-Vb, as shown in Fig. 3A and B. The detailed description of these 2 variants was provided in Materials and Methods. Both variants were also evaluated on the *DeepPPISP task*. Figure 3C depicts the performance comparison between EnsemPPIS and its 2 variants. Obviously, EnsemPPIS demonstrated superior performance compared to EnsemPPIS-Va and EnsemPPIS-Vb across all metrics, particularly in terms of MCC and AUPRC. This suggested that the ensemble of the 2 separately trained base models was more effective compared to the approach of initially integrating the 2 base models and training them simultaneously.

Furthermore, we additionally assessed the performance of TransformerPPIS using different types of features or feature combinations, namely, ProtBERT, PSSM [49], DSSP [50], and One-hot [22], and the results were depicted in Fig. 3D and E. Consequently, BERT-based feature outperformed the traditional handcrafted features on MCC and AUROC, and the inclusion of ProtBERT feature significantly enhanced the predictive performance.

(b) *Performance evaluation on DELPHI task*

EnsemPPIS was further assessed on *DELPHI task*, as shown in Table 2. Due to the unavailability of structural information in the training data, the evaluation and comparison of methods in this task focused solely on those utilizing protein sequences. This allowed for a fair and direct assessment of the performance of sequence-based methods in predicting PPI sites. All results were calculated by using the source code or web server. As a result, EnsemPPIS proved to be the best method. Specifically, considerable improvements in F1, AUPRC, and MCC were achieved by 5.8%, 8.8%, and 4.7%, respectively, compared with the SOTA method DELPHI.

**Table 2.** Comparison of the predictive performance of EnsemPPIS and other state-of-the-art methods on *DELPHI task*. All comparison methods use only protein sequences and are sorted alphabetically. The best results are shown in bold.

Method	ACC	F1	AUROC	AUPRC	MCC
DELPHI ^a^	**0.848**	0.364	0.746	0.326	0.278
DLPred ^b^	0.835	0.308	0.724	0.272	0.214
SCRIBER ^b^	0.838	0.322	0.719	0.275	0.230
SPRINGS ^a^	0.811	0.211	0.608	0.178	0.103
EnsemPPIS	0.821	**0.385**	**0.770**	**0.354**	**0.291**

^a^ Results obtained by reproducing the source code. ^b^ Results obtained by utilizing the web server.

In summary, EnsemPPIS achieved remarkable improvements in residue-level prediction of PPI sites using only protein sequences, outperforming all existing sequence-based methods and comparable to even the most advanced structure-based methods. In this study, protein sequences were input into the pretrained language model ProtBERT to obtain the protein embeddings. Currently, there are some large protein language models that are able to generate informative latent vectors for residues [51], such as ESM-2 [52] and AminoBERT [53]. These models utilize advanced deep learning techniques and large-scale training data to capture intricate features and patterns within protein sequences. By comprehensively leveraging these large language models, it is indeed possible to further enhance the performance of EnsemPPIS.

### Broader applicability of EnsemPPIS using only primary sequences

EnsemPPIS was also evaluated and compared on the *GraphPPIS task*, and the results can be found in Table S2. Two additional methods using protein structures, namely, RGN and GraphPPIS, were reproduced and evaluated in this task. All results were calculated using the source code or web server. Several methods compared on *DeepPPISP task* were not included in the *GraphPPIS task* for comparison, such as EGRET, because they were not provided with the training source code, thus preventing their retraining. As a result, EnsemPPIS once again outperformed all sequence-based methods and even achieved better performance than some structure-based approaches. Specifically, considerable improvements in F1, AUPRC, and MCC were achieved by 7.5%, 10.3%, and 17.2%, respectively, compared with the best existing method using protein sequences. In addition, EnsemPPIS also surpassed 2 of the structure-based methods (SPPIDER and DeepPPISP) on F1, AUPRC, and MCC, but slightly lagged behind RGN and GraphPPIS.

Although EnsemPPIS is inferior to RGN and GraphPPIS in the *GraphPPIS task* and only comparable to EGRET in the *DeepPPISP task*, it promises to be an indispensable tool and is applicable for the whole proteome, because it is free from the inherent limitations of structure-based methods, namely, the acute dependence on precise protein structures and the improper use of protein conformation for model training.

**Fig. 4.**
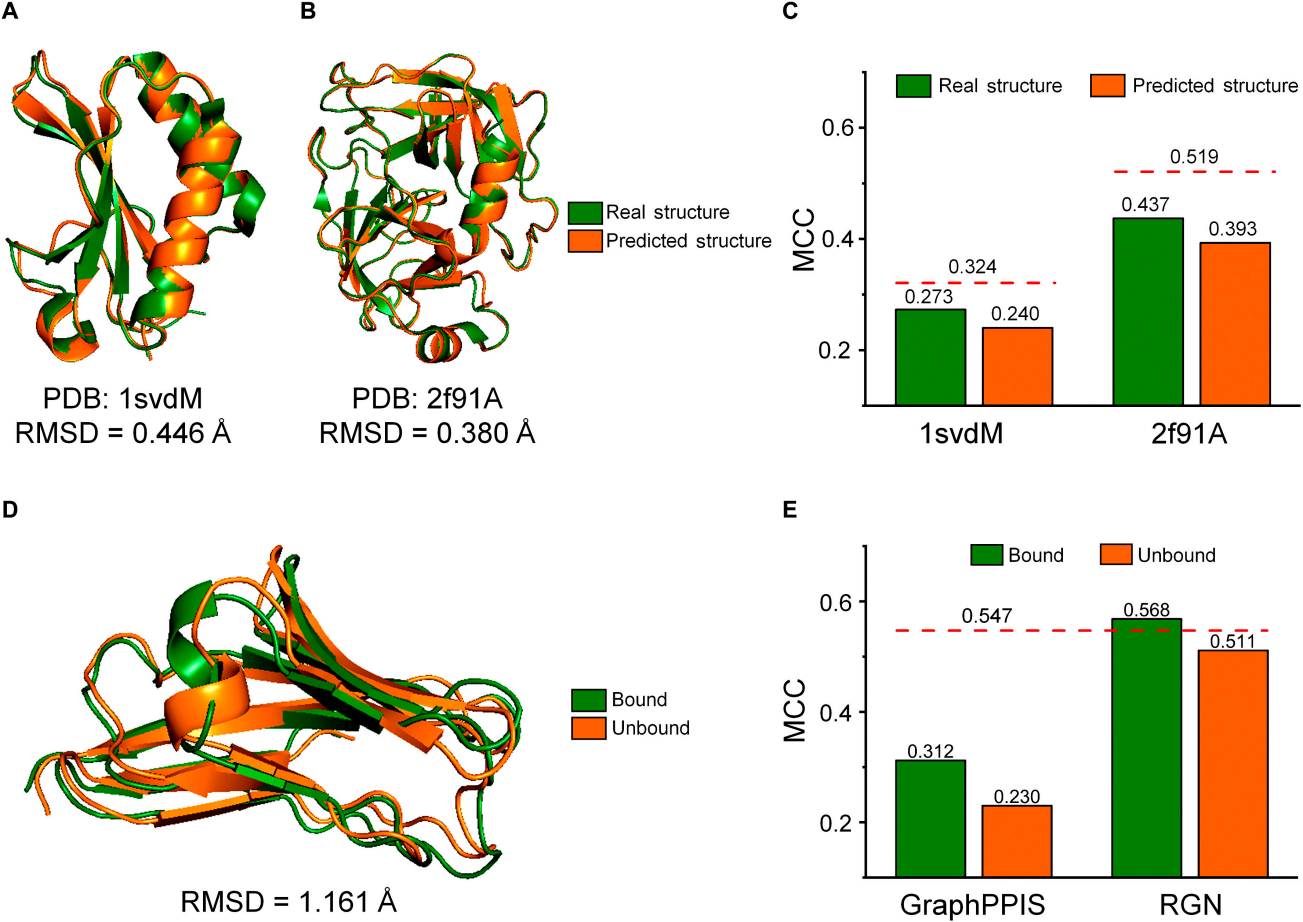
The impact of predicted structures and conformational changes on the performance of structure-based methods. (A) Real structure of the protein (PDB: 1svdM) and structure predicted by AlphaFold2. (B) Real structure of the protein (PDB: 2f91A) and structure predicted by AlphaFold2. The AlphaFold2 predictions are colored in orange and overlaid on the ground truth (green). (C) Performance of EnsemPPIS and EGRET on 1svdM and 2f91A using real structures and predicted structures. Green bars represent the MCC values of EGRET using the real structures, and orange bars represent the MCC values of EGRET using the predicted structures. The red dashed lines denote the MCC values of EnsemPPIS using only primary sequences. (D) Bound (PDB: 1qa9B) and unbound conformations (PDB: 1ci5A) of the same protein (UniProt: P19256). The bound conformation is colored in green and overlaid on the unbound conformation (orange). (E) Performance of EnsemPPIS, GraphPPIS, and RGN using the bound and unbound conformations. Green bars represent the MCC values of GraphPPIS and RGN using the bound conformation, and orange bars represent the MCC values using the unbound conformation. The red dashed line denotes the MCC value of EnsemPPIS using only primary sequence.

The first limitation of structure-based methods is that the lack of experimentally validated protein structures severely limits their scope of application [43,54]. This limitation can be partially alleviated through the use of advanced protein structure prediction tools such as AlphaFold2 [55,56], RoseTTAFold [57], ESMFold [52], and RGN2 [53]. To investigate the impact of predicted protein structures on the performance of structure-based methods, we tested the performance of EGRET on Test70 dataset using the structures predicted by AlphaFold2. The results showed that the predictive accuracy on many proteins decreased to varying degrees while using predicted structures in place of real structures. As shown in Fig. 4A and B, the AlphaFold2 predictions were colored in orange and overlaid on the ground truth (green). AlphaFold2 made accurate predictions for 2 proteins from RCSB Protein Data Bank (PDB) (PDB: 1svdM and PDB: 2f91A), with root mean square deviation (RMSD) of 0.446 and 0.380 Å, respectively [58]. Unfortunately, even with predicted structures of such high accuracy (RMSD value lower than 1.0 Å [55]), EGRET’s predictive performance for both proteins declined significantly. As illustrated in Fig. 4C, the MCC of two proteins achieved by EGRET decreased by 0.033 and 0.044, respectively, when the predicted structures were used as input, indicating that structure-based methods are highly sensitive to slight structural errors. Notably, due to the identical protein sequence between real structure and predicted structure, EnsemPPIS was not affected by any structural errors in predicting PPI sites and outperformed EGRET on both proteins in terms of MCC (the red dashed line in Fig. 4C). Moreover, currently available protein structure prediction methods have some significant limitations, particularly regarding the prediction of structures for proteins with low homology or missense mutations [59–64]. These inaccurate protein structure predictions will seriously mislead the results of structure-based PPI sites prediction approaches.

Protein conformation undergoes changes when binding with a partner [44,65,66], and currently available structure-based PPI sites prediction tools were typically trained using protein complex structures, which limits their accuracy and generalization ability when predicting PPI sites on unbound-form proteins [35]. To elaborate the second limitation of structure-based methods, we compared the predictive performance of RGN and GraphPPIS on the same protein with different conformations (i.e., bound and unbound conformations). The human lymphocyte function-associated antigen 3 (UniProt: P19256) in Test60 dataset was randomly selected as a case to conduct this analysis. As shown in Fig. 4D, the RMSD value between bound conformation (PDB: 1qa9B) and unbound conformation (PDB: 1ci5A) was 1.161 Å, and the position of α-helix (or β-sheet) in the 2 conformations was different, indicating that conformational changes indeed occurred during the binding process. As expected, both RGN and GraphPPIS presented an obvious decrease in MCC when predicting PPI sites on unbound conformation, as displayed in Fig. 4E. This suggested that models trained with complex structure information are limited in their robustness and generalization ability when making predictions on monomeric protein structures. PPI sites prediction methods that solely rely on protein sequences are not subject to the limitation of conformational changes because protein sequences remain consistent across different conformations. This offers an advantage in scenarios where accurate structural information is not readily available or when dealing with proteins with dynamic conformations. Specifically, EnsemPPIS exhibited noteworthy performance on both bound and unbound conformations, achieving the MCC value of 0.547 in both scenarios (as shown by the red dashed line in Fig. 4E). Importantly, this performance surpassed that of RGN and GraphPPIS specifically on the unbound conformation. In summary, our proposed EnsemPPIS overcomes the limitations associated with structure-based methods by solely relying on the information derived from primary protein sequences, and holds great advantages of broader applicability and stronger generalization ability.

### Superior performance of EnsemPPIS in protein-level prediction

(a) *EnsemPPIS outperforms SOTA ensemble learning method*

**Fig. 5.**
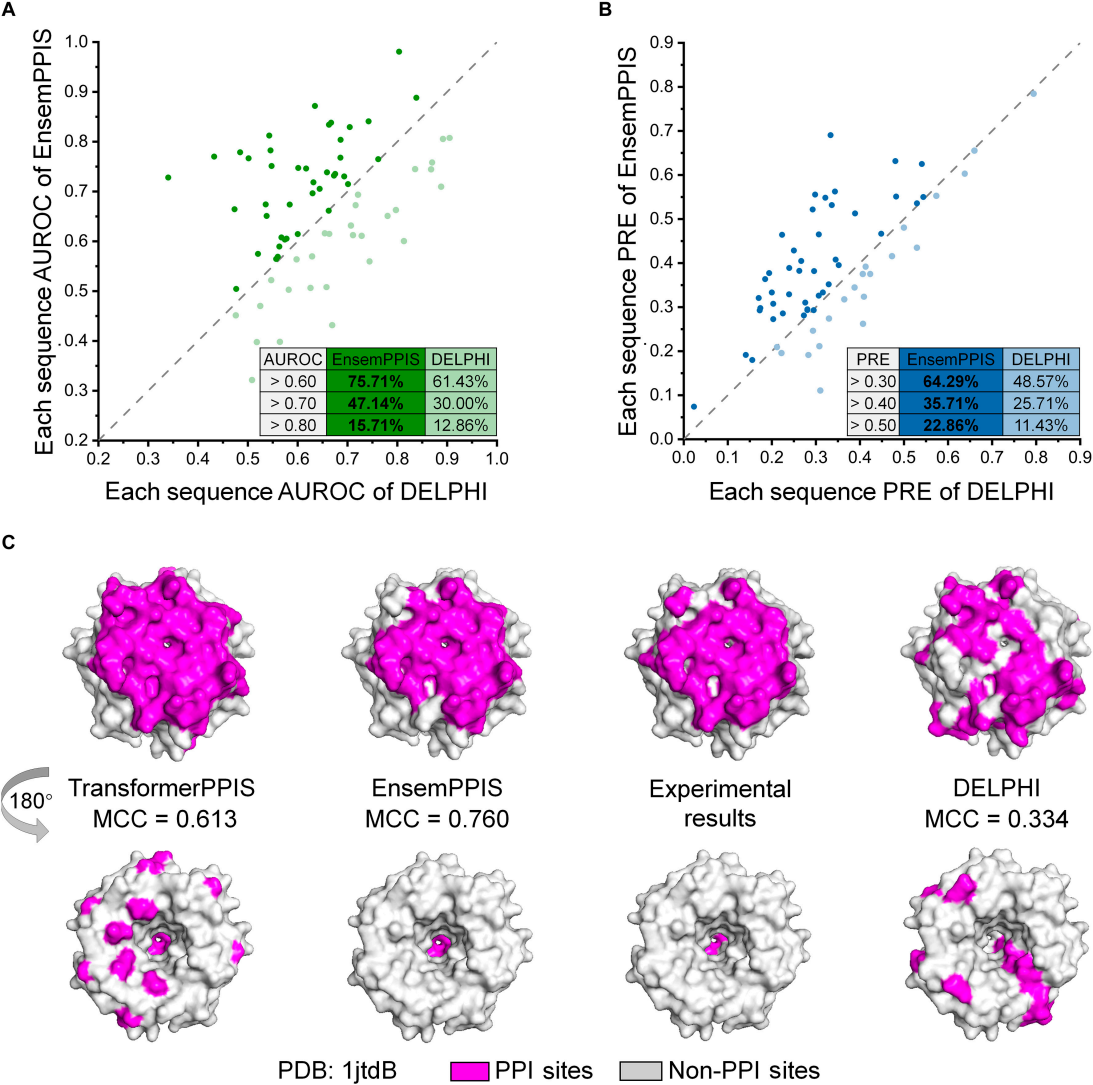
The comparison of EnsemPPIS and DELPHI in protein-level prediction on the Test70 dataset. (A) AUROC comparison between EnsemPPIS and DELPHI. (B) PRE comparison between EnsemPPIS and DELPHI. DELPHI is the current state-of-the-art ensemble method for the prediction of PPI sites using protein sequences. The scatter plot shows the performance comparison between EnsemPPIS and DELPHI, where each scatter represents a protein sequence in the Test70 dataset. The proportions of proteins across different intervals of AUROC and PRE are noted in the table. (C) Visualization of the prediction results achieved by TransformerPPIS, EnsemPPIS, and DELPHI for a specific protein (PDB: 1jtdB). PPI sites are shown in purple, and non-PPI sites are shown in gray.

EnsemPPIS consistently demonstrated superior performance in predicting PPI sites at the residue level. However, it is worth noting that similar predictive methods are commonly employed for individual protein predictions in downstream research. Therefore, we further assessed the performance of EnsemPPIS in protein-level prediction on the *DeepPPISP task*. We conducted a comparative analysis between our method and the SOTA ensemble learning method DELPHI to evaluate their performance in predicting individual protein sequences from the Test70 dataset. The results of this comparison were depicted in Fig. 5. Specifically, DELPHI only learned local and global sequential features based on convolutional neural network (CNN) and recurrent neural network (RNN), respectively. As a result, EnsemPPIS achieved protein predictions with AUROC values exceeding 0.60, 0.70, and 0.80 at rates of 75.71%, 47.14%, and 15.71%, respectively (as shown in Fig. 5A), and it predicted proteins with PRE values exceeding 0.30, 0.40, and 0.50 at rates of 64.29%, 35.71%, and 22.86%, respectively (as shown in Fig. 5B). EnsemPPIS outperformed DELPHI in terms of predicting a greater number of proteins with superior AUROC or PRE values across various intervals.

To elucidate the advantage of EnsemPPIS in predicting individual proteins, 2 specific proteins (PDB: 1jtdB and PDB: 1b6cA) were randomly selected as cases to visualize the prediction results of TransformerPPIS, EnsemPPIS, and DELPHI. As shown in Fig. 5C, the PPI sites on protein 1jtdB predicted by DELPHI exhibited a relatively dispersed pattern, whereas the PPI sites predicted by TransformerPPIS were more spatially concentrated, predominantly distributed on the same surface of the protein. This indicated that TransformerPPIS might learn the local structure of protein based on its sequence and capture the information about residues close in space. Furthermore, by rotating the protein conformation, as shown at the bottom of Fig. 5C, it was obvious that EnsemPPIS further reduced the false-positive rate, thereby enhancing the predictive performance (MCC = 0.760). The visualization of the prediction results for the protein 1b6cA was depicted in Fig. S1. Similar observations can be made, suggesting that EnsemPPIS attained the highest level of MCC (MCC = 0.542) while effectively managing the false-positive rate. This was attributed to the integration of GatCNNPPIS base model, which was capable of learning local sequential features.

(b) *EnsemPPIS is robust on sequences of different lengths*

Existing sequence-based methods predominantly focused on local sequential features of residues, largely neglecting the sequence interdependency [22]. This oversight tended to compromise the performance of these methods when predicting long sequences due to the critical role of long-range residue interactions in the formation of PPI [23,35]. As reported by DeepPPISP, the protein length greatly impacted the predictive performance and its performance significantly deteriorated when predicting longer sequences [22].

**Fig. 6.**
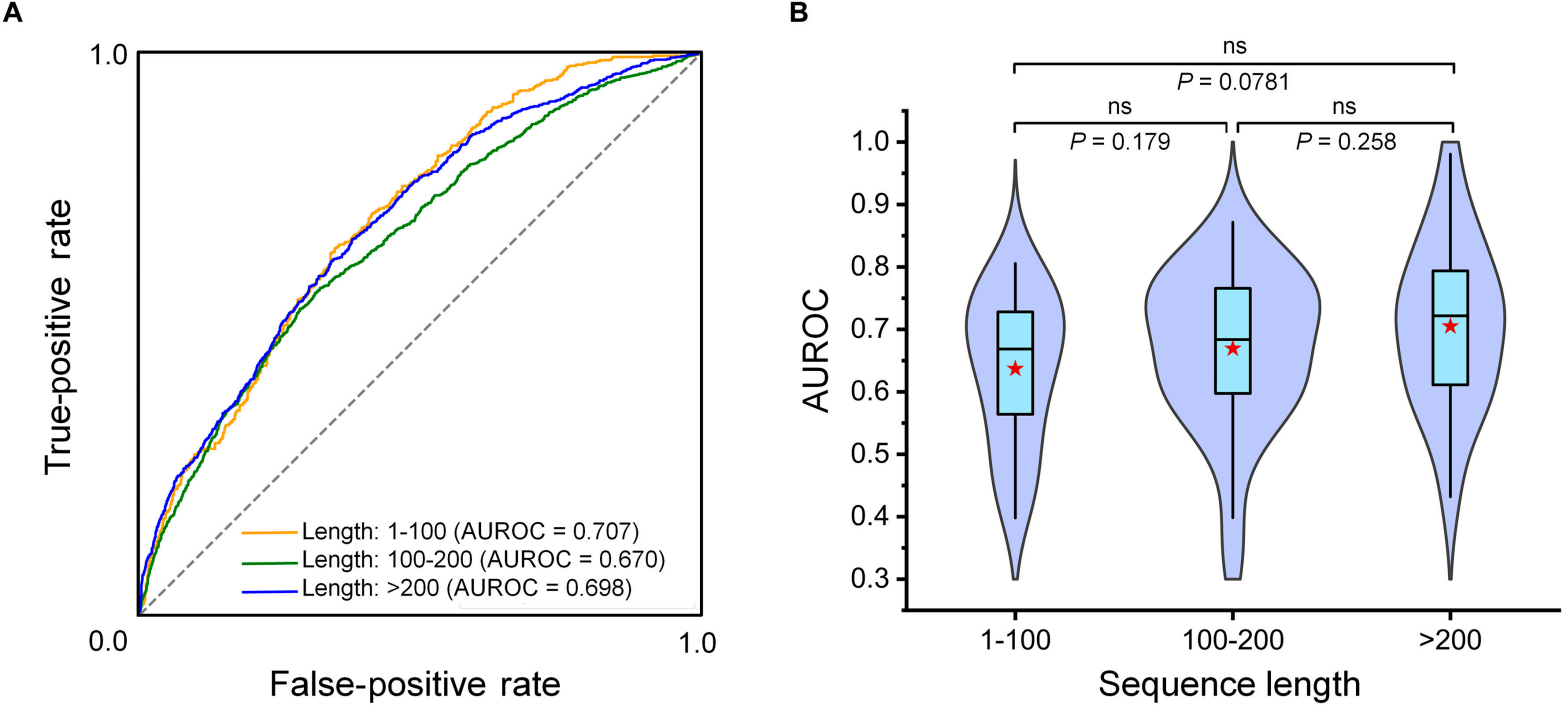
Performance evaluation of EnsemPPIS on different sequence lengths in both residue-level and protein-level prediction tasks on the Test70 dataset. (A) Receiver operator characteristic curve (ROC) and AUROC of EnsemPPIS at the residue level for different sequence lengths. The yellow, green, and blue curves represent the ROC of sequences with short length (1 to 100 residues), medium length (100 to 200 residues), and long length (>200 residues), respectively. (B) Distributions of each sequence AUROC achieved by EnsemPPIS at the protein level under different length categories. The box bounds the interquartile range divided by the median, with whiskers extending to 1.5 times the interquartile range. Each red star represents the mean. Each violin plot illustrates the kernel probability density, where the shaded area represents the proportion of the samples located there. The Mann–Whitney *U* test is used to perform the statistical analysis and calculate *P* values, and all *P* values are 2-sided.

Therefore, we also evaluated the predictive performance of EnsemPPIS on sequences of varying lengths in the Test70 dataset. All the 70 sequences were grouped into 3 categories, namely, short length (less than 100 residues), medium length (100 to 200 residues), and long length (more than 200 residues). The number of sequences of short length, medium length, and long length was 18, 32, and 20, respectively. We evaluated EnsemPPIS on different lengths in both residue-level and protein-level prediction tasks. As illustrated in Fig. 6A, at the residue level, EnsemPPIS exhibited similar AUROCs in predicting PPI sites from sequences of varying lengths. In addition, Fig. 6B displays the distributions of each sequence AUROCs achieved by EnsemPPIS in predicting proteins from different length categories at the protein level. EnsemPPIS maintained consistent predictive performance across proteins of varying lengths (*P* > 0.05) according to the Mann–Whitney *U* test [67]. The results indicated the robustness of EnsemPPIS in predicting proteins of different lengths, which might be attributed to the ability of TransformerPPIS in capturing long-range residue interactions from sequences.

### Pattern analysis based on the interpretability of EnsemPPIS

**Fig. 7.**
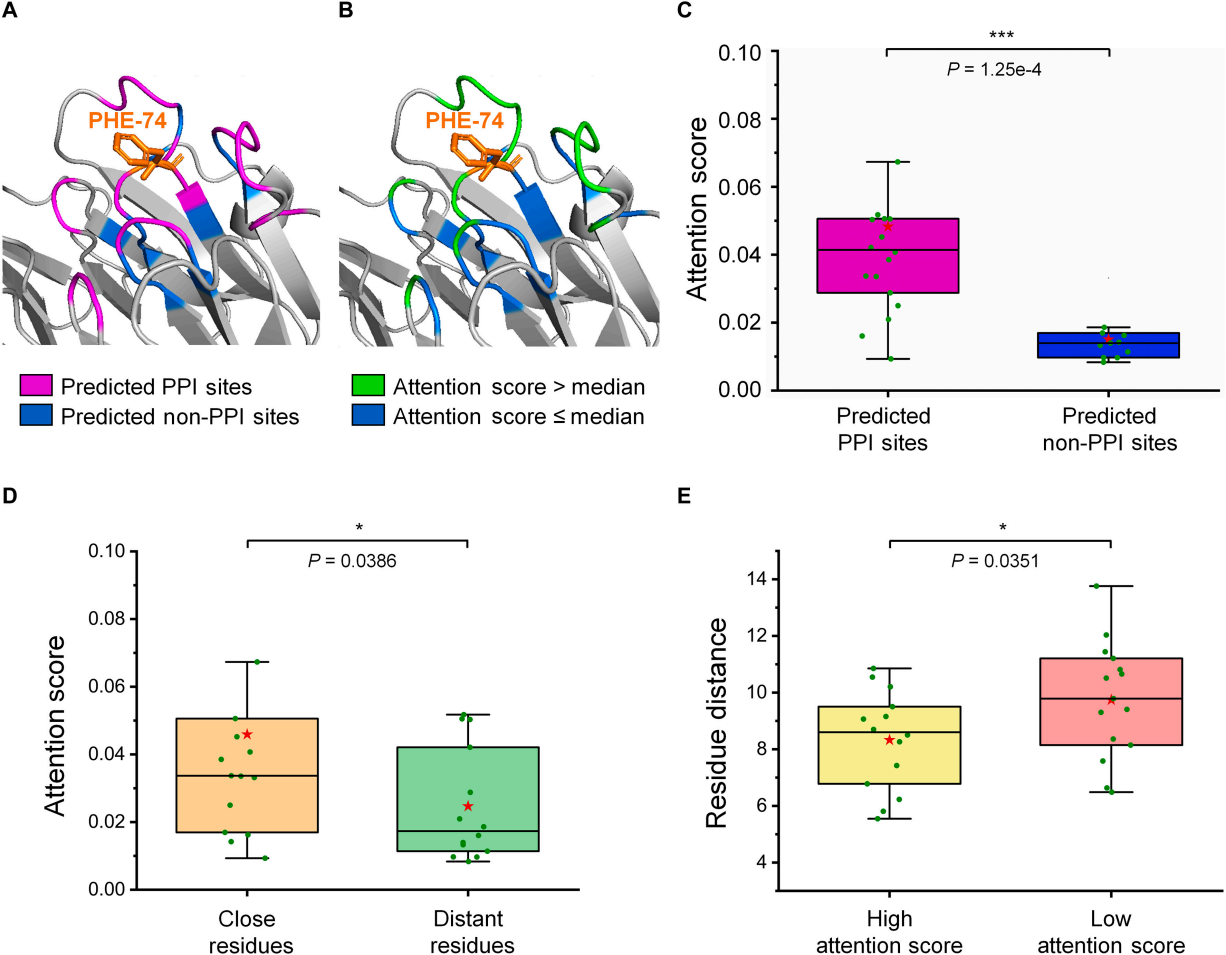
Attention analysis of residues within the 8 Å of the PPI site PHE-74 on a specific protein (PDB: 1jtdB) based on the predicted labels and the spatial distances to PHE-74. (A) Visualization of residue distribution based on their predicted labels. The predicted PPI sites are denoted in purple, and the predicted non-PPI sites are denoted in blue. (B) Visualization of residue distribution based on their attention scores. All the residues are divided into 2 groups according to the median of the attention scores. Residues with higher attention scores are shown in green, while those with lower attention scores are shown in blue. (C) Boxplot of attention scores for residues predicted as PPI sites and non-PPI sites. (D) Boxplot of attention scores for close residues and distant resides. Residues with distance less than or equal to the median value are labeled as “Close Residues,” and the remaining residues are labeled as “Distant Residues.” (E) Boxplot of residue distances for residues with high attention score and with low attention scores. Residues with attention score higher than or equal to the median value are categorized into “High Attention Score,” and the remaining residues are categorized into “Low Attention Score.” The box bounds the interquartile range divided by the median, with whiskers extending to 1.5 times the interquartile range. Each red star represents the mean value. The Mann–Whitney *U* test is used to perform the statistical analysis and calculate the *P* value.

The black box nature of deep learning methods calls for careful investigation of interpretability [68–70]. Owing to the implementation of the self-attention mechanism, the TransformerPPIS base model of EnsemPPIS exhibited commendable interpretability. Inspired by EGRET [23], the residue PHE-74 on the PDB protein 1jtdB was selected for the in-depth pattern analysis based on the interpretability of TransformerPPIS. We used the Spearman rank-order correlation [23] to calculate the correlation coefficient between the attention scores and predicted labels of residues within different distance ranges. As shown in Table S3, within the range of 5, 6, and 8 Å, the attention scores assigned to residues consistently exhibited a significant positive correlation with the predicted labels (all *P* < 0.05). In the case of the 8-Å range, the correlation coefficient (*r*) was calculated to be 0.697, with a corresponding *P* value of 2.71 × 10^−5^. To gain further insights, we divided the residues within this range into 2 groups based on either the median of the attention scores or the predicted labels. This division allowed us to visualize the distribution of residues and examine their characteristics. As depicted in Fig. 7A and B, within the range of 8 Å, residues predicted as PPI sites (purple residues in Fig. 7A) significantly overlapped with those with higher attention scores (green residues in Fig. 7B). Figure 7C reveals that residues predicted as PPI sites had notably higher attention scores than those predicted as non-PPI sites (*P* = 1.25 × 10^−4^) according to the Mann–Whitney *U* test.

PPI sites are relatively aggregated in protein structures, and local structural features play a crucial role in the formation of PPI. This implies that the interactions among residues within local structures play a crucial role in predicting PPI sites, and therefore, structure-based methods conduct the prediction by learning the features from spatially proximate amino acids [35]. However, spatially close residues may be distant in sequence. This poses a challenge for existing sequence-based methods, as they primarily emphasize the local sequential features of PPI sites. Consequently, capturing long-range residue interactions becomes difficult within the framework of these methods. In this study, the TransformerPPIS module was able to extract residue interactions including long-range interactions based on primary sequences. Again, take the residue PHE-74 as an example, its surrounding residues within the 8-Å range can be divided into 2 groups according to their distances to PHE-74 or attention scores. Specifically, we first defined the residue distance based on the average distance of all atoms between 2 residues. A total of 15 residues with distance less than or equal to the median value were grouped into “Close Residues,” while the remaining 14 residues were grouped into “Distant Residues.” The Mann–Whitney *U* test was then employed to examine the significant difference of the attention scores between these 2 groups. As illustrated in Fig. 7D, the *P* value was 0.0386, indicating that the attention scores of “Close Residues” were significantly higher than those of “Distant Residues.” Similarly, these residues were classified into another 2 groups, namely, “High Attention Score” and “Low Attention Score,” based on the median of their attention scores. As shown in Fig. 7E, the *P* value was 0.0351, which implied that residues with higher attention scores were spatially closer in local structure, but might be far apart in sequence. This pattern analysis suggested that residues closer in local space contributed more to the formation of PPI sites, which corroborated the fact that residues closer in space interact more significantly [71].

In summary, these findings highlighted that the TransformerPPIS base model within EnsemPPIS is fully capable of learning residue interactions, particularly the long-range interactions within the local structure of PPI sites using only primary sequences. This capability allows the model to extract meaningful connections between protein sequences and structures, ultimately leading to improved performance in predicting PPI sites.

### Availability of EnsemPPIS web server

A web server that implements EnsemPPIS was constructed in this study, which is convenient for researchers to apply our proposed PPI sites prediction method. The EnsemPPIS server was deployed on a Linux server of an Intel Xeon Gold 6149 3.10GHz CPU with 8 cores and 64 GB of memory based on the Python web framework of Django. As an open online platform, all users could freely access it through popular web browsers, including Google Chrome, Mozilla Firefox, Safari, and Internet Explorer 10 (or later).

EnsemPPIS requires only the FASTA-formatted protein sequences as input, and users should set a project name to associate their PPI sites prediction task. After successful submission, the information necessary to schedule the task would be placed into a MySQL database. Users could find their submitted project displayed on the “Queue” page of the web server. Clicking on the corresponding task information bar will redirect users to the program processing page, which offers 2 key functions: (a) encoding the input protein sequences using the pretrained ProtBERT and providing a downloadable *pickle* file containing the embedding vectors; (b) identifying potential PPI sites on all protein sequences and making a downloadable text file containing the prediction results. EnsemPPIS is freely available at http://idrblab.org/ensemppis.

## Conclusion

In this study, to improve the accuracy of PPI sites prediction and expand the application scope, a novel transformer-based ensemble learning method for PPI sites prediction, EnsemPPIS, was proposed, which incorporated 2 base models, namely, TransformerPPIS and GatCNNPPIS. EnsemPPIS was designed to extract residue interactions by leveraging the transformer and integrate global and local sequential features through ensemble learning. EnsemPPIS exhibited leading performance across multiple tasks, surpassing all existing sequence-based prediction methods and demonstrating its broader applicability in comparison to structure-based methods. Additionally, EnsemPPIS exhibited superior and robust performance in both residue-level and protein-level prediction tasks. Moreover, pattern analysis based on the interpretability of EnsemPPIS revealed its ability to learn residue interactions directly from protein sequences. EnsemPPIS is expected to facilitate in-depth understanding of molecular biology and advance research of drug discovery.

## Materials and Methods

### Benchmark datasets and evaluation metrics

In this study, the performance of our proposed EnsemPPIS was comprehensively assessed on 3 PPI sites prediction tasks, including *DeepPPISP task* [22], *GraphPPIS task* [35], and *DELPHI task* [34]. The basic information about the datasets used in the 3 tasks is described below, and Table S1 provides the statistics of these datasets.

#### (a) *DeepPPISP task*

The Train352 and Test70 datasets used in the *DeepPPISP task* were obtained from DeepPPISP [22]. The DeepPPISP dataset was generated by combining 3 widely used benchmark datasets, namely, Dset_186 [72], Dset_72 [72], and PDBset_164 [22], each collected from the PDB database [73] and built through a data filtering process involving 6 steps [72]. In total, there were 422 protein sequences in the DeepPPISP dataset, each with the resolution less than 3.0 Å and sequence homology lower than 25%. A surface amino acid was defined as a PPI site if its absolute solvent accessibility decreases by at least 1.0 Å^2^ upon protein binding [74]. For a fair comparison, we used the same data splitting scheme as DeepPPISP [22]. Thus, the training dataset Train352 contained 352 protein sequences and the independent test dataset Test70 was composed of 70 protein sequences. A subset of Train352 with 50 hold-out proteins is further randomly selected to form the validation dataset. As a result, there were 302 proteins in the training dataset, 50 proteins in the validation dataset, and 70 proteins in the test dataset.

#### (b) *GraphPPIS task*

The Train335 and Test60 datasets used in the *GraphPPIS task* were originally constructed by GraphPPIS and were also obtained by integrating the 3 datasets mentioned above (Dset_186, Dset_72, and PDBset_164) [35]. After the fusion of 3 benchmark datasets, BLASTClust [75] was further applied to remove protein sequences with similarities over 25%, leaving 395 nonredundant proteins. Subsequently, 335 proteins were randomly picked as the training data (Train335), and the remaining 60 proteins were used as the independent test data (Test60). To ensure a fair comparison, the Train335 and Test60 datasets used in this study were consistent with those used by GraphPPIS.

#### (c) *DELPHI task*

The Train9982 and Test355 datasets in *DELPHI task* were collected by DELPHI, a recent research of PPI sites prediction using sequences [34]. The Test355 dataset was a subset of Dset_448 dataset [32], which was built based on the BioLip database [76] and consisted of 448 nonredundant proteins with pairwise similarities lower than 25%. In the Dset_448 dataset, the interaction sites in a protein complex were defined as the residues to which 2 atoms belonged, based on a distance criterion. Specifically, if the distance between 2 atoms from different chains was found to be less than 0.5 Å plus the sum of their Van der Waals radii, these residues were identified as interacting sites. To ensure the comparability with another competing method named DLPred [77], the developers of DELPHI removed 93 proteins sharing similarities above 40% with any sequences in DLPred’s training dataset, and then constructed the Test355 dataset. To obtain the Train9982 dataset, the developers collected a large dataset from a previous study [78] and used PSI-CD-HIT [79] to remove sequences sharing similarities over 25% with any sequences in the Test355, followed by the removal of sequences with similarities above 25% among the remaining proteins. Among 9,982 sequences in the Train9982 dataset, 1,110 sequences were randomly selected to compose the validation dataset and the remaining sequences were utilized to train the model. It is important to note that the Train9982 dataset cannot be applied directly to train structure-based PPI sites prediction methods for the lack of structural information. Therefore, several methods using only sequences were evaluated in this task.

The prediction of PPI sites is essentially a binary classification task. In this study, the interaction sites were taken as positive samples and non-interaction sites as negative samples. To fully evaluate the performance of EnsemPPIS and other competing methods, 7 widely used evaluation metrics were adopted in this study, including accuracy (ACC), precision (PRE), recall (REC), F1-score (F1), Matthews correlation coefficient (MCC), area under the receiver operator characteristic curve (AUROC), and area under the precision–recall curve (AUPRC). All metrics were calculated using the Scikit-learn package [80], and the formulas for computing these metrics were provided in Supplementary Methods. Serious data imbalance is reported to be a significant characteristic of PPI sites datasets, making MCC, F1, and AUPRC the most important and comprehensive indicators as they can emphasize more on the minority class [22,81,82].

### Deep learning architecture of EnsemPPIS

To convert protein sequences into embeddings, the pretrained protein language model, ProtBERT, was used to generate an *L* × 1,024 matrix for each protein sequence, where *L* is the sequence length and each amino acid is represented by a 1,024 embedding vector. ProtBERT is a BERT model pretrained on UniRef100 through self-supervised learning, which can capture biophysical features of protein sequences [48,82,83]. The embeddings of proteins were further passed to the 2 base models of EnsemPPIS, namely, TransformerPPIS and GatCNNPPIS. Inspired by the great ability of transformer in extracting sequence features, the novel TransformerPPIS was proposed for predicting PPI sites using the modified transformer. The architecture of TransformerPPIS, as shown in Fig. 2, consists of 3 modules: the encoder, the decoder, and the classifier module.

#### (a) *Encoder module*

In contrast to the original transformer framework, the encoder of TransformerPPIS uses a gated convolutional network with Conv1D and gated linear unit in place of the self-attention layers [84]. Conv1D mainly captures the contextual representation of residues with local biases and learns the global protein features by assembling local features of all residues. The gated linear unit can enhance the network's capacity to process nonlinear information and extract more informative representations from proteins. The sequence embedding of a protein is first converted into an *L* × 64 matrix using the FC and then fed into the gated convolutional network. The hidden layers *h*_0_, …, *h_l_* in the gated convolutional network are computed as Eq. 1:

$$h_{l}\left(X\right)=\left(X*W_{1}+b_{1}\right)⊗\sigma \left(X*W_{2}+b_{2}\right)$$ (1)

where ***X*** ∈ *ℝ*^*n*×*m*_1_^ is the input of layer *h_l_*; ***W***_1_ ∈ *ℝ*^*k*×*m*_1_×*m*_2_^, ***W***_2_ ∈ *ℝ*^*k*×*m*_1_×*m*_2_^, *b*_1_ ∈ *ℝ*^*m*_2_^, and *b*_2_ ∈ *ℝ*^*m*_2_^ are trainable parameters; *l* is the number of encoder layers; *n* is the length of the sequence; *m*_1_ and *m*_2_ are the dimension of input and hidden features of the gated convolutional network, respectively; *k* is the kernel size of Conv1D; *σ* is the sigmoid function; and ⨂ represents the element-wise product between matrices [84]. In this study, *l* is 3, *m*_1_ is 64, *m*_2_ is 128, and *k* is 7. The encoder module adopts residual connection and layer normalization to solve the oversmoothing problem [85]. The output of encoder, an *L* × 64 matrix, is the final representation of a protein.

#### (b) *Decoder module*

The decoder module of TransformerPPIS is specifically designed to learn and capture residue interactions within protein sequences. The input of decoder module contains 2 parts: the global feature of the protein output by the encoder module and the original embedding of a specific residue obtained by ProtBERT. The decoder module mainly consists of multi-head self-attention layers and feedforward layers. The multi-head self-attention layer extracts the interactions between the specific residue and other residues, which takes 3 inputs: the queries, ***Q***; the keys, ***K***; and the values, ***V*** [86,87]. TransformerPPIS regards the residue embedding as ***Q*** and the global protein feature as ***K*** and ***V***, and calculates the attention weight using ***Q*** and ***K***. The calculation formula is as follows:

$$\text{attention}\left(Q,K,V\right)=\text{softmax}\left(\frac{QK^{T}}{\sqrt{d_{k}}}\right)V$$ (2)

where *d_k_* is a scaling factor depending on the dimension of the hidden layer. The mask operation in the original transformer framework is modified in the decoder module to ensure that the complete sequence information is accessible. Accurately identifying PPI sites necessitates careful attention to the features of the local structure surrounding these sites [40]. However, residues that are spatially close may be far apart in sequence due to the intricate folding patterns and 3-dimensional arrangement of protein structures. The self-attention mechanism employed in TransformerPPIS empowers the model to effectively capture the interactions between remote residues in a protein sequence. Another major component of decoder module is the feedforward layer, which improves the expressiveness of features by nonlinear transformation [88]. After each self-attention layer and feedforward layer, the residual connection and layer normalization are used.

#### (c) *Classifier module*

The output of decoder module is the interaction feature between the specific residue and the global protein sequence. The interaction feature vector is further fed to the classifier module, which is composed of 3 FCs and the ReLU activation function [89]. Finally, the probability of a residue being a PPI site is calculated by the softmax function.

The GatCNNPPIS base model presented here can be viewed as a simplified version of TransformerPPIS, consisting solely of the encoder and the classifier modules. In the output of the encoder module (the *L* × 64 matrix), each vector represents the local contextual feature of a particular residue. GatCNNPPIS takes this vector as input and directly feeds it into the classifier module, which outputs the probability of the corresponding residue being an interaction site.

### Model training and hyperparameter tuning

The classification of PPI sites poses a challenge due to the inherent imbalance in the dataset. After the softmax function normalized the output of the network into the probability over the 2 classes (interaction site and non-interaction site), the weighted cross-entropy loss function was adopted to compute the loss values of samples, which were subsequently used to calculate the gradient of parameter update in the backward propagation process [90]. The weighted cross-entropy loss function assigned different class weights to positive and negative samples, allowing the model to prioritize the minority class and allocate more attention to its predictions. During model training, the ratio between the weights of positive and negative samples was determined based on the model’s performance on the validation dataset. Specifically, in the *DeepPPISP task* and *GraphPPIS task*, the weight ratio was set to 5:1, while in the *DELPHI task*, it was set to 3:1. The LookAhead optimizer and RAdam optimizer were used during the training process [91]. In each PPI sites prediction task, the EnsemPPIS used the same training scheme as that of the competing method [22,34,35]. Specifically, in the *DeepPPISP task* and *DELPHI task*, the training dataset was used to train EnsemPPIS, and the validation dataset was used to evaluate the predictive performance and optimize the hyperparameters, followed by the assessment and report of the performance of the best model on the independent test dataset. In the *GraphPPIS task*, the 5-fold cross-validation was performed on the training dataset to avoid the influence of random errors, that is, all proteins in the Train335 dataset were randomly divided into 5 folds. Among these 5 folds, 4 folds were utilized to train EnsemPPIS and the remaining fold served as the validation dataset to evaluate the model. This procedure was repeated 5 times, with each fold serving as the validation dataset. The average of the 5 evaluation results was then calculated to obtain the overall evaluation result. Based on this result, the best hyperparameters were selected. When the hyperparameters were determined, the final model was trained using all training data and evaluated on the independent test dataset. The early stopping strategy was applied to reduce overfitting and training cost [92–94]. In order to facilitate the convergence of training and improve the capacity of generalization, regularization methods including dropout and weight decay were used during training EnsemPPIS [95–97].

As an ensemble learning framework, the 2 base models of EnsemPPIS (TransformerPPIS and GatCNNPPIS) were separately trained using the same training procedure. To optimize EnsemPPIS, we selected the optimal combinations of base models [98]. After the completion of model training, the 2 saved models were loaded for individual prediction of PPI sites. In addition, we constructed 2 variants of EnsemPPIS to evaluate the outcomes achieved by combining the 2 base models into a single model for concurrent training. The architectures of the 2 variants were depicted in Fig. 3A and B. Specifically, in the first variant of EnsemPPIS (EnsemPPIS-Va), the output of TransformerPPIS’s decoder and the output of GatCNNPPIS’s encoder were concatenated. The concatenated vector was then fed into multiple FCs to obtain the probability of being PPI site. In the second variant (EnsemPPIS-Vb), the output of TransformerPPIS’s decoder and the output of GatCNNPPIS’s encoder were separately passed through 3 FCs. The resulting 2-dimensional vectors were then concatenated, and the concatenated 4-dimensional vector was further processed through an FC to obtain the predicted probability. The output of each variant was utilized to calculate the loss for jointly updating the parameters of the 2 base models.

Three most influential hyperparameters (batch size, learning rate, and dropout rate) were tuned according to the predictive performance on the validation dataset. As a result, the optimal combination of the above 3 hyperparameters was decided (batch size = 128, learning rate = 0.0005, dropout rate = 0.1). All the hyperparameter settings of EnsemPPIS were summarized in Table S4. EnsemPPIS was implemented with Pytorch 1.2.0 (http://pytorch.org/) and supported distributed training [99]. All scripts were written by Python 3.7.11, and all models were developed on the computer with Intel Xeon Gold 6132 CPU @ 2.60GHz, NVIDIA Tesla P100 16GB GPU and 263GB RAM on CentOS Linux release 7.9.2009 (Core).

### A variety of methods compared with EnsemPPIS

A comprehensive review on the previously published tools for PPI sites prediction was conducted in this study, which were systematically compared with our proposed EnsemPPIS, as shown in Table S5. These methods can be grouped into sequence-based and structure-based depending on whether the protein structural information is used. Sequence-based methods include ISIS [100], PSIVER [72], SPRINGS [31], RF_PPI [27], SCRIBER [32], DELPHI [34], ProNA2020 [33], and DLPred [77]. SCRIBER used a 2-layer architecture to perform partner type-specific prediction of protein-binding residues [32]. ProNA2020 utilized the combination of homology-based inference and machine learning methods to predict protein-macromolecular binding residues using only protein sequences [33]. DELPHI was the SOTA sequence-based method that used 12 feature groups to encode proteins, and incorporated CNN and RNN with the ensemble learning strategy to enhance its predictive performance [34]. Structure-based methods include SPPIDER [28], IntPred [21], DeepPPISP [22], EGRET [23], GraphPPIS [35], and RGN [40]. DeepPPISP proposed an end to end deep learning model, which used CNN to combine local contextual and global features for PPI sites prediction [22]. EGRET constructed an edge aggregated graph attention network to effectively leverage protein structural information [23]. GraphPPIS employed evolutionary information and structural properties of amino acids to train the deep convolutional network for the prediction of PPI sites [35]. RGN applied PSSM, hidden Markov model, hydrogen bond estimation algorithm, and ProtBERT for node representation and constructed a residue-based graph attention and convolutional network [40].

## Data Availability

The EnsemPPIS web server is freely available at http://idrblab.org/ensemppis, with all source codes and benchmark datasets in this study. The trained models and the EnsemPPIS standalone source code can be found at https://github.com/idrblab/EnsemPPIS.

## Supplementary Materials

Supplementary 1 Methods Tables S1 to S5 Fig. S1 References
