# Supplementary material for: A Transformer-Based Ensemble Framework for the Prediction of Protein–Protein Interaction Sites
Source: Research (Wash D C). 2023 Sep 27;6:0240. doi: 10.34133/research.0240 (PMC10528219; doi:10.34133/research.0240)
Supplement: Supplementary 1 — Methods Tables S1 to S5 Fig. S1 References [file research.0240.f1.docx]

**Supplementary Materials for:**

**A transformer-based ensemble framework for the prediction of protein-protein interaction sites**

Minjie Mou**^1^**^,^†, Ziqi Pan**^1^**^,^†, Zhimeng Zhou**^1^**, Lingyan Zheng**^1^**, Hanyu Zhang**^1^**, Shuiyang Shi**^1^**, Fengcheng Li**^1^**, Xiuna Sun**^1^**, and Feng Zhu**^1^**^,^**^2^**^,^*

**^1^** College of Pharmaceutical Sciences, The Second Affiliated Hospital, Zhejiang University School of Medicine, National Key Laboratory of Advanced Drug Delivery and Release Systems, Zhejiang University, Hangzhou 310058, China

**^2^** Innovation Institute for Artificial Intelligence in Medicine of Zhejiang University, Alibaba-Zhejiang University Joint Research Center of Future Digital Healthcare, Hangzhou 330110, China.

† These authors contributed equally to this work.

* Address correspondence to: zhufeng@zju.edu.cn

**Running title**: *A novel ensemble framework for PPI sites prediction*

# Supplementary Methods

**Evaluation Metrics**

Seven widely used evaluation metrics were adopted in this study, including accuracy (ACC), precision (PRE), recall (REC), F1-score (F1), Matthews correlation coefficient (MCC), area under the receiver operator characteristic curve (AUROC) and area under the precision-recall curve (AUPRC). The formulas for computing these metrics are as follows:

$$\begin{aligned} \mathrm{ACC}=\frac{TP+TN}{TP+TN+FP+FN}\#\left( AUTONUM \backslash* Arabic \right) \end{aligned}$$

$$\begin{aligned} \mathrm{PRE}=\frac{\mathrm{TP}}{TP+FP}\#\left( AUTONUM \backslash* Arabic \right) \end{aligned}$$

$$\begin{aligned} \mathrm{REC}=\frac{\mathrm{TP}}{TP+FN}\#\left( AUTONUM \backslash* Arabic \right) \end{aligned}$$

$$\begin{aligned} F1=\frac{2\times PRE\times REC}{PRE+REC}\#\left( AUTONUM \backslash* Arabic \right) \end{aligned}$$

$$\begin{aligned} \mathrm{MCC}=\frac{TP\times TN-FP\times FN}{\sqrt{\left( TP+FP \right)\times\left( TP+FN \right)\times\left( TN+FP \right)\times\left( TN+FN \right)}}\#\left( AUTONUM \backslash* Arabic \right) \end{aligned}$$

where $\mathrm{TP}$, $\mathrm{TN}$, $\mathrm{FP}$ and $\mathrm{FN}$ stand for the number of true positives, true negatives, false positives and false negatives, respectively.

**Supplementary Table S1**. Statistics of benchmark datasets used in multiple PPI sites prediction tasks.

| **Task** | **Dataset** | **No. Proteins** | **No. PPI Sites** | **No. Non-PPI Sites** |
| --- | --- | --- | --- | --- |
| *DeepPPISP task* | Train352 | 352 | 11,079 | 62,102 |
|  | Test70 | 70 | 2,332 | 9,459 |
| *GraphPPIS task* | Train335 | 335 | 10,374 | 55,992 |
|  | Test60 | 60 | 2,075 | 11,069 |
| *DELPHI task* | Train9982 | 9982 | 427,687 | 3,826,511 |
|  | Test355 | 355 | 11,467 | 84,473 |

The columns give, in order, the task names, dataset names, the number of proteins in each dataset, the number of PPI sites, and the number of non-PPI sites in each dataset.

**Supplementary Table S2**. Comparison of the predictive performance of EnsemPPIS and other state-of-the-art methods on *GraphPPIS task*.

| **Method** | **ACC** | **F1** | **AUROC** | **AUPRC** | **MCC** |
| --- | --- | --- | --- | --- | --- |
| DeepPPISP ^b^ | 0.657 | 0.335 | 0.653 | 0.276 | 0.167 |
| DELPHI ^b^ | 0.706 | 0.373 | 0.699 | 0.319 | 0.227 |
| DLPred ^a^ | 0.700 | 0.357 | 0.677 | 0.294 | 0.205 |
| GraphPPIS ^b^ | 0.772 | 0.431 | 0.681 | **0.487** | 0.307 |
| ProNA2020 ^a^ | 0.738 | 0.326 | - | - | 0.176 |
| RGN ^b^ | **0.813** | **0.455** | **0.779** | 0.429 | **0.344** |
| SCRIBER ^a^ | 0.667 | 0.350 | 0.665 | 0.278 | 0.193 |
| SPPIDER ^a^ | 0.735 | 0.220 | 0.511 | 0.193 | 0.062 |
| EnsemPPIS | 0.716 | 0.401 | 0.726 | 0.352 | 0.266 |

^a^ Results obtained by utilizing the web server. ^b^ Results obtained by reproducing the source code. ProNA2020 only makes binary predictions and its AUROC and AUPRC are not calculated. DeepPPISP, GraphPPIS, RGN and SPPIDER use protein structural information. DELPHI, DLPred, ProNA2020 and SCRIBER use protein sequences. EnsemPPIS is proposed in this study. All comparison methods are sorted alphabetically. The best results are shown in bold.

**Supplementary Table S3**. The correlation analysis results between the attention values and predicted labels of residues around the PHE-74 in the protein (PDB: 1jtdB). The residues with distance to PHE-74 less than 5 Å, 6 Å and 8 Å were analyzed. The results were obtained using the Spearman rank-order correlation.

| **Distance to PHE-74** | **Number of Residues** | **Correlation Coefficient (*r*)** | ***P*-value** |
| --- | --- | --- | --- |
| 5 Å | 12 | 0.666 | 0.0181 |
| 6 Å | 17 | 0.603 | 0.0104 |
| 8 Å | 29 | 0.697 | 2.71e-5 |

**Supplementary Table S4**. The hyperparameter settings of EnsemPPIS.

| **Hyperparameter** | **Value** |
| --- | --- |
| Number of encoder layers | 3 |
| Number of decoder layers | 3 |
| Number of attention heads | 8 |
| Kernel size of Conv1D in encoder layers | 7 |
| Dimension of hidden features | 64 |
| Dimension of hidden features in feedforward layers | 256 |
| Dropout rate | 0.1 |
| Learning rate | 0.0005 |
| Learning rate decay | 1 |
| Weight decay | 0.0001 |
| Batch size | 128 |
| Patience in early stopping | 10 |

**Supplementary Table S5**. The detailed information of various methods for PPI sites prediction. ACH, averaged cumulative hydropathy; CNN, convolutional neural network; GAT, graph attention network; GCN, graph convolutional network; GNN, graph neural network; LR, linear regression; LDA, linear discriminant analysis; LSTM, long short-term memory; NB, naïve Bayes; NN, neural network; PRSA, predicted relative solvent accessibility; PSSM, position specific scoring matrix; RF, random forest; RNN, recurrent neural network; RSA, relative surface accessibility; SVM, support vector machine.

| **Method** | **Feature** | **Algorithm** | **URL of Source Code or Web Server** | **Ref.** |
| --- | --- | --- | --- | --- |
| SPPIDER ^a^ | single sequence-based attributes, features derived from evolutionary profiles of protein families, features based on protein tertiary structure, RSA prediction-based fingerprints | SVM, NN and LDA | http://sppider.cchmc.org | [1] |
| ISIS | evolutionary profiles, predicted secondary structure and solvent accessibility | NN | Not available | [2] |
| PSIVER | PSSM; predicted accessibility | NB | Not available | [3] |
| SPRINGS | PSSM; ACH; PRSA | NN | https://sites.google.com/site/predppis/ | [4] |
| RF_PPI | 20 features (DynaMine score, RSA, accessible surface area, entropy and length of query sequence) | RF | http://www.ibi.vu.nl/downloads/RF_PPI/ | [5] |
| IntPred ^a^ | 11 features (sequence and structural features) | RF and NN | https://github.com/ACRMGroup/intpred/ | [6] |
| SCRIBER | 1090 features | LR | http://biomine.cs.vcu.edu/servers/SCRIBER/ | [7] |
| DeepPPISP ^a^ | PSSM, secondary structure, sequence one-hot | CNN | https://github.com/CSUBioGroup/DeepPPISP | [8] |
| DELPHI | 12 feature groups | CNN and RNN | https://github.com/lucian-ilie/DELPHI | [9] |
| EGRET ^a^ | ProtBERT | GNN | https://github.com/Sazan-Mahbub/EGRET | [10] |
| ProNA2020 | predicted secondary structure, PRSA, bio-physical properties of amino acids | SVM, ProtVec and NN | https://www.predictprotein.org | [11] |
| DLPred | PSSM, physical properties, hydropathy index, etc. | LSTM | http://qianglab.scst.suda.edu.cn/dlp/ | [12] |
| GraphPPIS ^a^ | PSSM, HMM, DSSP | GCN | https://github.com/biomed-AI/GraphPPIS | [13] |
| RGN ^a^ | PSSM, HMM, DSSP, ProtBERT | GCN and GAT | https://github.com/demainchen/RGN | [14] |

^a^ Uses protein structural information.


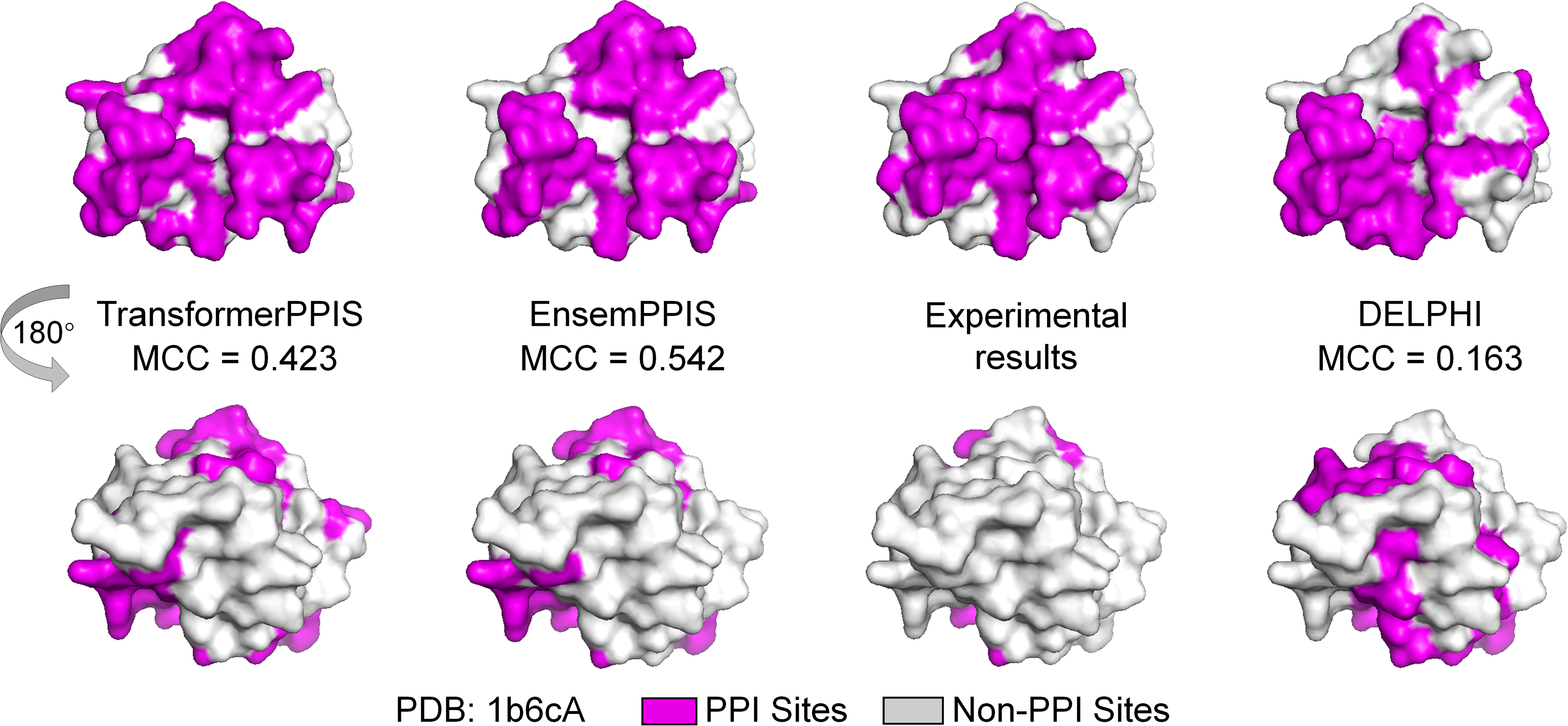


**Supplementary Figure S1**. Visualization of the prediction results achieved by TransformerPPIS, EnsemPPIS and DELPHI for a specific protein (PDB: 1b6cA). DELPHI is the current state-of-the-art ensemble method for the prediction of PPI sites using protein sequences. PPI sites are shown in purple, and non-PPI sites are shown in grey.

# References

1. Porollo A, Meller J. Prediction-based fingerprints of protein-protein interactions. *Proteins*. 2007;66(3):630-645.

2. Ofran Y, Rost B. ISIS: interaction sites identified from sequence. *Bioinformatics*. 2007;23(2):e13-e16.

3. Murakami Y, Mizuguchi K. Applying the Naive Bayes classifier with kernel density estimation to the prediction of protein-protein interaction sites. *Bioinformatics*. 2010;26(15):1841-1848.

4. Singh G, Dhole KD, Pai P, Mondal SK. SPRINGS: Prediction of Protein- Protein Interaction Sites Using Artificial Neural Networks. *J Proteomics Computational Biol*. 2014;1(1):7.

5. Hou Q, De Geest PFG, Vranken WF, Heringa J, Feenstra KA. Seeing the trees through the forest: sequence-based homo- and heteromeric protein-protein interaction sites prediction using random forest. *Bioinformatics*. 2017;33(10):1479-1487.

6. Northey TC, Baresic A, Martin ACR. IntPred: a structure-based predictor of protein-protein interaction sites. *Bioinformatics*. 2018;34(2):223-229.

7. Zhang J, Kurgan L. SCRIBER: accurate and partner type-specific prediction of protein-binding residues from proteins sequences. *Bioinformatics*. 2019;35(14):i343-i353.

8. Zeng M, Zhang F, Wu FX, Li Y, Wang J, Li M. Protein-protein interaction site prediction through combining local and global features with deep neural networks. *Bioinformatics*. 2020;36(4):1114-1120.

9. Li Y, Golding GB, Ilie L. DELPHI: accurate deep ensemble model for protein interaction sites prediction. *Bioinformatics*. 2021;37(7):896-904.

10. Mahbub S, Bayzid MS. EGRET: edge aggregated graph attention networks and transfer learning improve protein-protein interaction site prediction. *Brief Bioinform*. 2022;23(2):bbab578.

11. Qiu J, Bernhofer M, Heinzinger M, Kemper S, Norambuena T, Melo F, Rost B. ProNA2020 predicts protein-DNA, protein-RNA, and protein-protein binding proteins and residues from sequence. *J Mol Biol*. 2020;432(7):2428-2443.

12. Zhang BZ, Li JY, Quan LJ, Chen Y, Lu Q. Sequence-based prediction of protein-protein interaction sites by simplified long short-term memory network. *Neurocomputing*. 2019;357:86-100.

13. Yuan Q, Chen J, Zhao H, Zhou Y, Yang Y. Structure-aware protein-protein interaction site prediction using deep graph convolutional network. *Bioinformatics*. 2021;38(1):125-132.

14. Wang S, Chen W, Han P, Li X, Song T. RGN: Residue-Based Graph Attention and Convolutional Network for Protein-Protein Interaction Site Prediction. *J Chem Inf Model*. 2022;62(23):5961-5974.
